# Ferroelectret-based Hydrophone Employed in Oil Identification—A Machine Learning Approach

**DOI:** 10.3390/s20102979

**Published:** 2020-05-24

**Authors:** Daniel R. de Luna, T.T.C. Palitó, Y.A.O. Assagra, R.A.P. Altafim, J.P. Carmo, R.A.C. Altafim, A.A.O. Carneiro, Vicente A. de Sousa

**Affiliations:** 1 Department of Communications Engineering, Federal University of Rio Grande do Norte (UFRN), Natal 59078-970, Brazil; vicente.sousa@ufrn.edu.br; 2 Department of Electrical Engineering, UFRN, Natal 59078-970, Brazil; 3 Electrical and Computer Engineering Department, São Carlos Engineering School, University of São Paulo, Av. Trabalhador São-carlense 400, São Carlos 13566-590, Brazil; olivato@usp.br (Y.A.O.A); jcarmo@sc.usp.br (J.P.C.); altafim@sc.usp.br (R.A.C.A.); 4 Computer Systems Department, Informatics Center, Federal University of Paraíba, Rua dos Escoteiros, João Pessoa 58051-900, Brazil; ruy@ci.ufpb.br; 5 Department of Physics, FFCLRP, University of São Paulo, Av. Bandeirantes 3900, Ribeirão Preto 14040-91, Brazil; adilton@usp.br

**Keywords:** ferroelectric, hydrophone, oil identification, supervised learning, acoustic

## Abstract

This work focuses on acoustic analysis as a way of discriminating mineral oil, providing a robust technique, immune to electromagnetic noise, and in some cases, depending on the applied sensor, a low-cost technique. Thus, we propose a new method for the diagnosis of the quality of mineral oil used in electrical transformers, integrating a ferroelectric-based hydrophone and an acoustic transducer. Our classification solution is based on a supervised machine learning technique applied to the signals generated by an in-home built hydrophone. A total of three statistical datasets entries were collected during the acoustic experiments on four types of oils. The first, the second, and third datasets contain 180, 240, and 420 entries, respectively. Eighty-four features were considered from each dataset to apply to two classification approaches. The first classification approach is able to distinguish the oils from the four possible classes with a classification error less than 2%, while the second approach is able to successfully classify the oils without errors (e.g., with a score of 100%).

## 1. Introduction

Transformers are fundamental equipment in electrical power systems; their main function is the adjustment of voltage levels, serving as a link between generation, transmission, and distribution of electrical energy. Due to their importance, it is common sense that these equipment are directly related to the continuity and quality of the electricity supply system. Therefore, periodical or online monitoring of their operating condition has become increasingly important [1,2].

A key component of power transformers that requires constant supervision is the mineral oil placed inside them. This oil, responsible for insulating the internal coils and provide a cooling agent, undergo deterioration due to electrical and thermal efforts, which in turn generates decomposition products that cause the occurrence of equipment failures. Over time, some of its physical and chemical properties, such as color, viscosity, and water content, vary due to environmental conditions and exposure to electric fields [3].

A significant number of studies have presented effective solutions for monitoring transformers, aiming predictive maintenance [1,4,5], and many of them focus on oil analysis [6,7,8,9]. Among the diagnostic techniques available to assess the isolation condition of the transformers, it is possible to mention the physical-chemical analysis, the analysis of dissolved gases, the optical analysis, the measurement of the degree of polymerization (DP), the measurement and analysis of furans by High Performance Liquid Chromatography (HPLC) and acoustic analysis. Two of these techniques are widely used in preventive programs, and they include the analysis of dissolved gas (DGA) in oil and the physical-chemical analysis.

Some studies address acoustic and ultrasonic analysis as a predictive technique in the transformers monitoring for the detection of moisture in transformers oil [10,11,12]. Normally, water is hardly dissolved in oil due to the polarity of the molecules and the hydrophobic characteristic of the oil. However, new or regenerated oil contains minimal amounts of water, which are measured in ppm (parts per million). In addition, over time, during operation, the humidity level may increase due to the degradation of polymeric materials and the absorption of external moisture [13]. Therefore, the detection of moisture in insulating oil is important and is necessary due to its harmful characteristics to oil and other insulating components. Among the damages caused by water, it is possible to mention the decrease of the dielectric resistance of the oil, acceleration of the cellulose aging (used as insulating coating internally in the transformers), and formation of bubbles when the equipment is exposed to high temperatures [14]. Such features have been explored to acquire the quality of the oil inside the transformers.

Therefore, in order to detect types of oils by using only acoustic analysis, we developed two machine learning classification approaches. These techniques are in widespread use in research and industrial communities. Thus, even though this framework was developed to classify types of oils by using supervised learning, the same approaches and techniques can be used in similar problems or in a totally new classification problem. However, our solution is mainly based on an in-home built thermoformed ferroelectret as an acoustic transducer, providing a very low-cost solution.

We organize the paper as follows. Section 2 discusses some relevant literature related to acoustic and ultrasonic analysis, and ways of discriminating liquids. Section 3 details and contextualizes the problem addressed by this work. Section 4 presents our experimental setup and details the main materials used. In Section 5, we discuss the proposed framework utilized, detailing the two classification approaches. The results and discussions are presented in Section 6. Finally, our final remarks and futures works are presented in Section 7.

## 2. Related Works

As previously mentioned, some research involving acoustic and ultrasonic analysis in the detection of oil in transformers has been done in the last few decades [10,11,12,15,16].

In the article by Tokitou and Shida [10], a detection system to discriminate water in oil is presented. The detection method adopts the difference in the propagation time of an ultrasonic wave in water and oil, for each characteristic temperature. When water and oil are heated, these propagation times change in reverse. The authors claim that, for this reason, the proposed system does not need to provide any previous absolute values database as a reference. Additionally, the authors suggest the possibility of detecting the water present in the oil of a transformer using the proposed method.

Chang-ping and collaborators [11] developed a method for detecting moisture in transformer oil based on the difference in ultrasonic transit time. The proposed methodology assumes that the ultrasonic speed of oil and water are similar, but there is a relatively large difference with ice. In this way, the oil-water samples are frozen for measurements. In the experiments, different samples (300 ml) of mixtures, prepared by the authors, of oil and water are used. The method consists of using two identical measuring cells, where two transducers are attached, one operating as a transmitter and the other as a receiver, separated by a fixed distance. A standard oil sample is measured in a cell, without adding water. In the other cell, different oil-water mixtures are tested. A sinusoidal signal pulse is emitted, which passes through the medium and is then detected by the receiver. The two signals are compared and the analysis is made based on the difference in the ultrasonic propagation time of the signals. The results show that the higher the water content in the oil, the lower the propagation speed in the medium, and the greater the difference in propagation time between a standard oil and an oil-water mixture. In the article, the authors do not reveal the transducer model or the frequency used in the experiments.

Tyuryumina, Batrak and Sekackiy [12] use the acoustic emission method (AE) as an online diagnostic technique to identify failures in power transformers. The proposed method is used to measure acoustic signals caused by impurities (water, cellulose, gas) in the transformer oil. The methodology consists of a signal generator, two piezoelectric transducers and a computer where the signals are processed via software. Initially, a comparison test is carried out between two oil samples, a new and an aged one, present in transformer tanks, to verify the sensitivity of the proposed method. To determine the influence of water, cellulose and gas on transformer oil, the authors add different concentrations of these to different samples of new oil and then measure the signal. The authors analyze the frequency spectrum of the signal (1 to 10 kHz) to obtain information about the condition of the transformer. According to the results obtained, water and cellulose influenced the quality of the transformer oil. However, the AE method is not sensitive to the determination of the gas phase in the transformer oil in the selected frequency range.

The article by Palitó et al. [15] suggests an ultrasonic system developed to investigate the presence of moisture in transformers oil. This system consists of a function generator, two ultrasonic sensors, acting as an emitter and the other as a receiver, an acoustic chamber and an oscilloscope. In the experiments, samples of 600 ml of water and different oil samples from transformers with different levels of degradation are used. The experiments are carried out at frequencies of 2.25, 3.5, 5, and 10 MHz. Measurements of the amplitude of the sinusoidal burst are made for each of the liquids tested and for each frequency. In this study, it was observed that the higher the water content in the samples, the greater the amplitude of the signal. The authors conclude that of the four frequencies studied, the one that best suits the study is the frequency of 5 MHz because it was this that presented the greatest discrepancy between the amplitudes of the samples of transformer oils. In addition to signal amplitude measurements, measurements of ultrasonic propagation speed were also performed. The authors declare, based on the results obtained, that as the oil has higher water content, the ultrasonic propagation speed is lower, as reported in the literature.

Recently, the authors of Reference [16] provide solutions for power transformer problems using machine learning. Kunicki and Wotzka [16] use energy patterns based on the discrete wavelet transform to detect partial discharges (PD), and in a second step, eight classes of various faults or anomalies. The proposed two-step classification method is tested with real-life measurements, providing results that exceeded 98% of classification accuracy. Although its high accuracy, it depends on the PD occurrence.

## 3. Problem Definition and Paper Contributions

As already mentioned, the condition of the oil considerably affects the performance and service life of the transformers. A combination of electrical, physical and chemical tests can be performed to measure the change in electrical properties, the extent of contamination and the degree of deterioration in the insulating oil. The results of these tests are used to establish preventive maintenance procedures, to avoid unscheduled stops, early failures, and to prolong the life of the equipment [17].

Traditionally, the physical-chemical analysis and the dissolved gas analysis (DGA) in oil are the two techniques widely used by the maintenance sectors of electricity systems as predictive solutions for monitoring the conditions of the power transformers immersed in insulating mineral oil (IMO) [7,8,18,19,20,21].

The **physical-chemical analysis** allows inferring the state of the IMO by laboratory analysis of IMO samples from a transformer in service. The main physical-chemical characteristics, or tests, used as parameters for the classification of IMO, are color, appearance, dielectric strength, water content, acidity index, interfacial tension, dielectric losses, and density [8]. Table 1 presents the reference values for starting the control of IMO in a new equipment. This table contains test limits for ensuring that the IMO, in equipment after the processing and standing time before energization, is dry, contains no excess particulate matter, and contains a minimum amount of dissolved gas [22].

The moisture or water content in the transformer oil is highly undesirable, as it negatively affects the oil’s dielectric properties, increasing the electrical conductivity and dissipation factor, and reducing its electrical resistance [23]. In addition, the water content of the insulation system accelerates the degradation of the insulation, decreases the cooling efficiency of the transformer, and causes the emission of bubbles at high temperatures. In transformer oil, water can be originated from the atmosphere or be produced by the deterioration of insulating materials (cellulose and oil) [4,14]. Usually, the water content in the oil, given in milligrams per kilogram (mg/kg) or part per million (ppm), is measured by the Karl Fischer coulometric titration method, in which reagents are used [22,24]. In new or regenerated oil, the amounts of water must be minimal. As a rule, the acceptable unit content for a new oil in new equipment is 20 ppm, whereas for oil in equipment (transformers and reactors) in operation, the limit value for corrective action is 35 ppm (both values for the equipment category up to 69 kV). As the equipment category increases, the moisture content allowed in the oil decreases [22].

**Dissolved Gas Analysis (DGA)** is the most used technique to monitor the performance of power transformers [18,20,25,26], and other electrical equipment containing oil. Using the DGA, it is possible to assess the operating condition of the equipment’s insulation since this technique is able to identify various types of gases, thus allowing the diagnosis of different types of failures. The formation of gases can occur due to the natural aging process and/or as a result of equipment failure, even if it is still in its incipient phase [8]. Generally, DGA is performed using gas chromatography, a traditional method, which provides acceptable results. The chromatographic analysis of the gases dissolved in the oil is done in three stages. First, the oil samples are collected from operating transformers and transported to the laboratory. Then, the extraction of the gases from the oil sample can be done by vacuum extraction, stripper extraction, and headspace sampling. After the extraction, the gases are analyzed using techniques for interpretation of DGA, for example, the key gas, Doernenburg ratio, Rogers ratio, IEC ratio, and Duval triangle methods [20,27].

Physical-chemical analysis and DGA are consolidated techniques and are widely used by concessionaires, however for these analyses to be carried out, it is necessary to collect the oil sample in the field so that it can be taken to the laboratory for further analysis. In some cases, for example, in substations located far from the laboratory, the time required to transport this sample for analysis can cause economic losses for utilities.

Another technique that can be applied in diagnosing the quality of IMO is acoustic analysis. It is a method based on the propagation of acoustic waves, commonly known as the acoustic emission method (AE). This sensitive technique employs acoustic sensors to detect acoustic waves (continuous or pulsed) that are transferred to the environment [28,29]. The acoustic waves are then monitored according to acoustic parameters such as the speed of propagation, attenuation, acoustic impedance, and these parameters can be related to some physical properties of the environment, such as density, viscosity and elasticity. One of the advantages of this technique is that it can be used to indirectly evaluate variables of industrial and research processes in a non-destructive way, with the possibility of non-invasive and online applications [30].

The present paper provides a low-cost solution to detect the quality of IMO by using machine learning and acoustic analysis from signals collected by an in-home built acoustic transducer. Our key contributions are:

- a non-invasive acoustic method technique for diagnosing the IMO quality without dependence on partial discharges events;
- a preventive, local and fast-diagnosing technique complementary to a high-cost and offline Physical-chemical IMO quality analysis;
- a low-cost solution for IMO quality evaluation based on an in-home built acoustic transducer;
- an analysis of real-life transformer’s IMO measurements, and the proposal of two classification approaches using machine learning to recognize IMO quality;
- an improved experimental setup compared to Reference [31].

## 4. Experimental Setup

**Figure 1:**
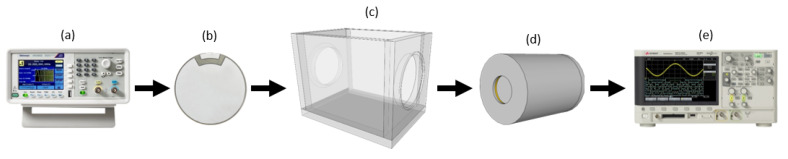
Experiments configuration scheme.

The experimental setup of our proof-of-concept evaluation is presented in Figure 1. The experimental setup comprises a function generator (a), an ultrasonic emitter (b), an acoustic chamber (c), an acoustic transducer (d) and an oscilloscope (e).

The function generator (a) is programmed to generate a sinusoidal signal in the SWEEP mode, feeding the ultrasonic emitter (b), which transmits the signal inside the acoustic chamber (c). Then, the acoustic transducer (d) receives the signal and feeds the digital oscilloscope. Finally, the signal is stored for further processing.

### 4.1. Ultrasonic Emitter

**Figure 2:**
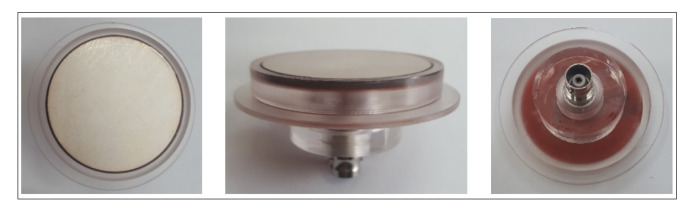
Ultrasonic emitter used.

Piezoelectric materials are increasingly popular and, due to their reduced cost, can be used as sensors and actuators in several scientific studies with a wide range of applications [2,31,32,33]. The ultrasonic emitter, shown in Figure 2, is a piezoelectric ceramic with a 50 mm in diameter, 2.6 mm thick, resonant frequency of 40 kHz, and power of 50 Watts. This ultrasonic ceramic is responsible for emitting the acoustic signal that will propagate in the liquid. To provide the electrical contact of this ceramic and connect it to the acoustic camera, an acrylic piece was developed, as also shown in Figure 2. The electrical connection was made through a female BNC connector, and the ultrasonic ceramic was fixed to the acrylic support using a high temperature sealing silicone.

### 4.2. Acoustic Chamber

**Figure 3:**
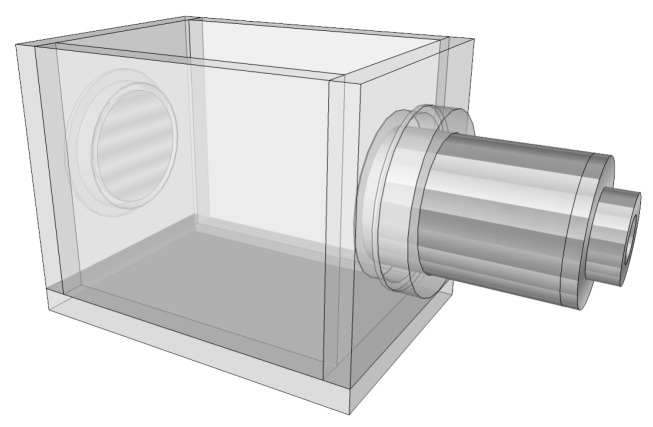
Illustration of the acoustic chamber prototype.

An acoustic chamber was developed to accommodate the ultrasonic emitter and the acoustic transducer. The prototype of the acoustic chamber was made in 10 mm crystal acrylic and had internal dimensions 80 × 100 × 120 mm in width, height and length, respectively. This prototype is illustrated in Figure 3 with the ultrasonic emitter and the acoustic transducer already attached.

### 4.3. Acoustic Transducer

**Figure 4:**
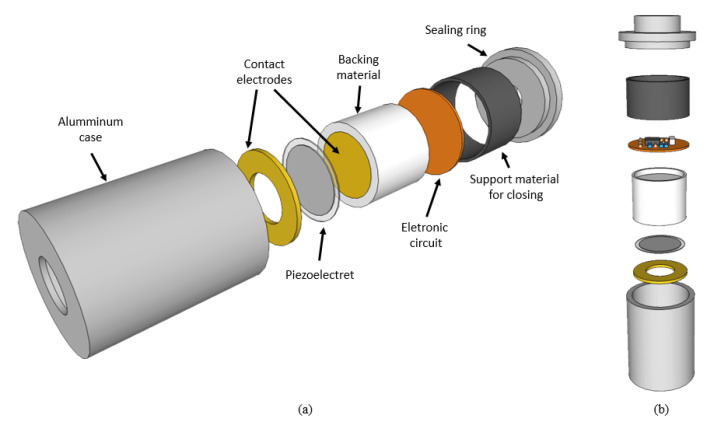
Acoustic transducer: design of the metallic enclosure, seen in perspective: (**a**) lateral; (**b**) frontal.

The main elements that make up the acoustic transducer consist of a metallic enclosure, an electronic pre-amplification circuit and a ferroelectret, which are presented in Figure 4. This proposal consists of an improvement of Prototype 1 presented in Palitó et al. [31] for liquid application purposes.

The metallic enclosure is responsible for the electrical shielding of the device and for the packaging of the amplifier and the ferroelectret sensor, the metallic electrodes and the pre-amplifier circuit board. The rear material, made of nylon, comprises the layer underlying the piezoelectric element and is responsible for dampening the vibration of the electromechanical film, which prevents reflections on the back of the active element and, consequently, avoids generating interference in the reception signal of the transducer.

**Figure 5:**
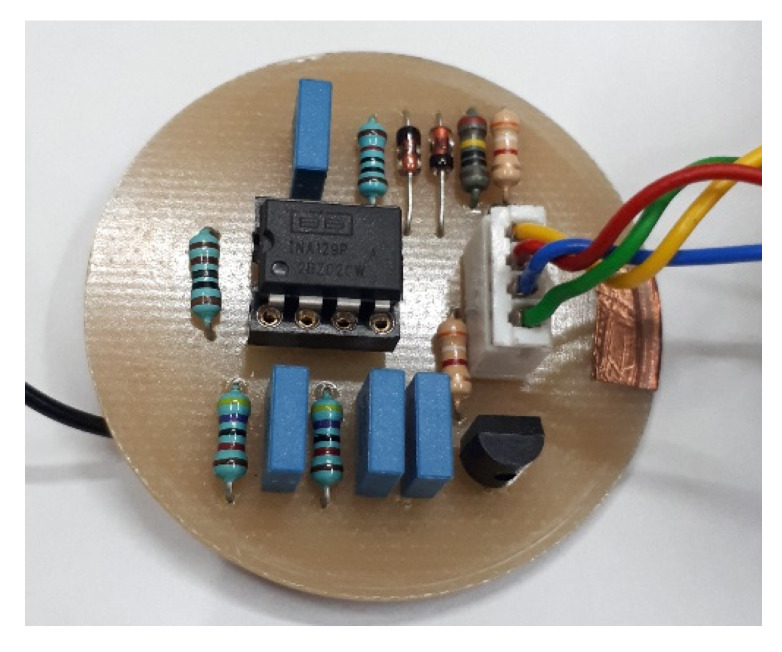
Electronic acoustic transducer circuit.

Inside the metallic enclosure, an electronic circuit composed of a preamplifier, a high-pass filter, and a differential amplifier is mounted. These three stages are presented in detail in Reference [31]. In order to increase the gain of the differential amplifier, we exchange the resistor $R_{G}=5.6\;k\Omega$ of the circuit presented in Reference [31], by a 1kΩ resistor. With this modification, the differential amplifier started to present a gain of 50.4 times (34.05 dB) and response in flat frequency up to 100 kHz. The complete amplification circuit (Figure 5) of this acoustic transducer has a final gain of 38.13 dB.

### 4.4. Ferroelectret

The ferroelectret that makes up the acoustic transducer is the result of the thermoformed piezoelectric technology, and it was produced with fluorinated ethylene-propylene (FEP) films and with open tubular channels. This technology was chosen due to previous results that showed stable piezoelectricity of 160 pC/N, at 80 °C [34].

**Figure 6:**
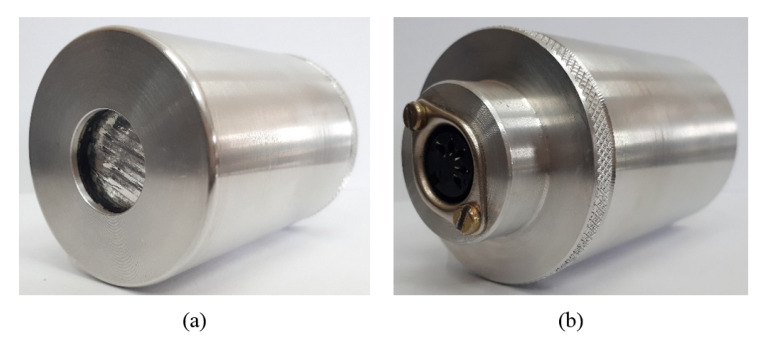
Acoustic transducer, seen in perspective: (**a**) frontal side; (**b**) and rear side.

The operating principles of the ferroelectret and its manufacturing process are described in detail in [34]. It consisted of laminating two 50 μm FEP films at 300 °C with a 100 μm polytetrafluoroethylene (PTFE) mold between them. The mold was designed to create a ferroelectret with equally spaced open tubular channels (1.5 mm wide and 100 μm high). The films were thoroughly cleaned with acetone before lamination to avoid grease or dust particles. After lamination, the PTFE mold was removed from the fused FEP layers, forming a two-layer polymeric structure with ten open channels. The polymeric structure was transformed into ferroelectret after circular aluminum electrodes were deposited on both sides of the structure, and a DC voltage of 3 kV was applied directly over the electrodes for 10 s. Figure 6 shows the acoustic transducer with the piezoelectric sensor used in experiments.

### 4.5. Measurement Methodology

**Figure 7:**
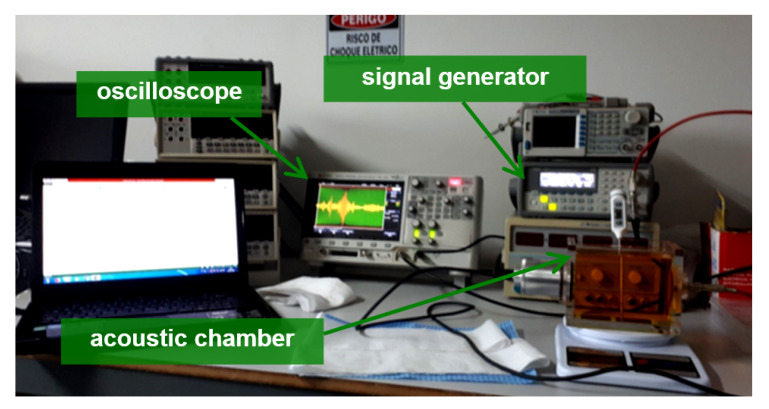
Experimental setup.

To drive the ultrasonic emitter a function generator (Agilent 33210A, 10 MHz) was used in SWEEP mode, with a sine wave of 10 V from peak to peak, covering a frequency range from 10 kHz to 100 kHz, linearly, over a 1 s time interval (in total 100.000 samples are generated). The function generator drives the acoustic transducer, causing it to transmit the low frequency ultrasound wave through the oils (800 ml) and producing an acoustic signal pattern due to multiple reverberations within the acoustic chamber. Subsequently, the acoustic transducer receives the signal and performs its function of converting the sound signal into an electrical signal and amplifying it. Finally, the signal is captured by the oscilloscope (Agilent Keysight DSOX2002A, 70 MHz, 2 GSa/s) where the signal is visualized and the data is saved with 100.000 samples. Figure 7 presents a photo of the experiment setup.

Two databases were used in our experiments, named as:

- **Database 1:** from oil samples collected in 2016;
- **Database 2**: with data samples collected in 2018.

**Figure 8:**
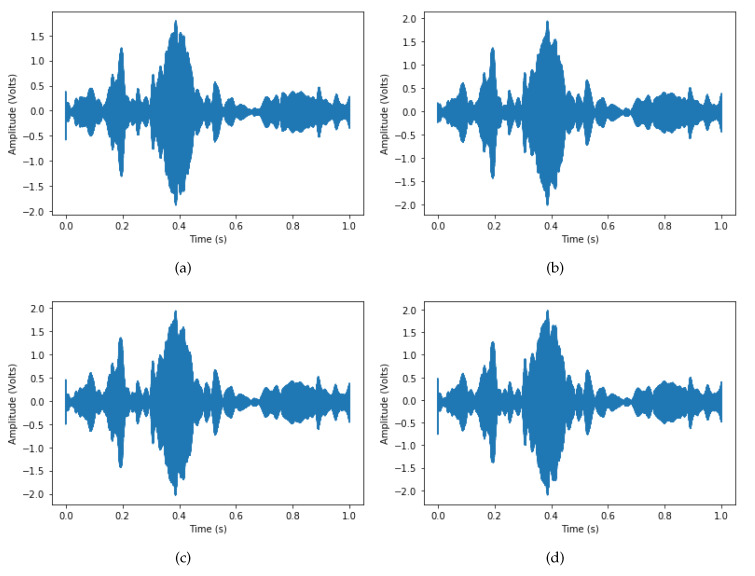
Example of Signal SWEEP for different types of oils: (**a**) new oil; (**b**) processed oil; (**c**) contaminated oil; and (**d**) out of service oil (Database 2).

In each database, there are oil samples with different levels of degradation, named in the following classes:

- New;
- Processed oil;
- Contaminated oil;
- Out of service oil;

These oil samples were collected from transformers under maintenance by the company Potencial, which provided the samples with the technical report of the physical-chemical analysis. The results of the physical-chemical analysis of the oil samples are presented in Table 2 and Table 3. The oils with suffixes 1 and 2 correspond to Databases 1 and 2, respectively. Figure 8 shows the SWEEP signal for the four different types of oils. As one can note, the behavior is similar, so that there is no way to classify the types of oils by a simple visual inspection. However, there are a number of differences regarding the amplitude when a specific time is set. In Section 5, we discuss how to use these responses as a way of classification the oils analyzed.

The experiments were repeated nine and ten times for each sample of transformer oils, then five and six measurements were made for different SWEEPs in each medium of Databases 1 and 2, respectively. Therefore, 45 and 60 measurements were taken for each transformer oil sample (new_1, processed_1, contaminated_1, out of service_1, new_2, processed _2, contaminated_2, and out of service_2), respectively.

## 5. Proposed Classification Framework

This section presents our proposed machine learning framework. The target problem focuses on classifying a SWEEP signal from the experiment to detect which class oil it comes from. The creation of the statistical dataset, as well as the classifiers used in this work, are detailed as follows.

### 5.1. Statistical Dataset

One of the greatest challenges in statistical learning is how to feed the learning machine with features and values that really can be used as part of the learning strategy [35]. On the other hand, we cannot use the SWEEP signal because it is a temporal series, instead, we calculated some general and moving statistical parameters that are important to our problem. For the former, we use the complete signal to get the measures, while the latter, we have to define a window, which is used to get the measures from that specific moving interval. For this paper, the statistical parameters calculated from the SWEEP signals include mean, moving mean, variance, moving variance, the difference between peaks and bottoms (Vpb), moving Vpb, correlation, and moving correlation. To calculate the moving statistical values, a window of 5.000 samples was defined. Thus, we created a dataset with these statistical values for all oil samples and established labels from which oil class the signals come from. This is an important step, once the classification problem is a type of supervised learning.

**Figure 9:**
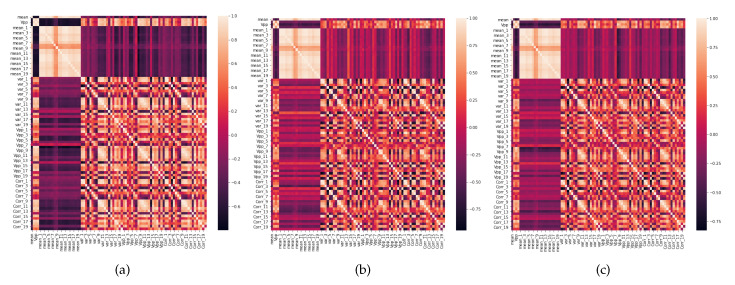
Correlation Heatmap: (**a**) Dataset 1, (**b**) Dataset 2, (**c**) Dataset 3.

In total, we have three datasets from the experiments made in 2016 (Database 1), 2018 (Database 2), and another with both databases together. Also, each dataset has 84 features with 180, 240 and 420 entries, respectively. Table 4 resumes the main configuration of the pre-processing phase of this proposed classification framework. In addition, Figure 9 shows the correlation heatmap of the features of the three datasets. Note that Figure 9a,b are very similar; in other words, the features behave in a very similar way, which means that in the classification phase, the same features (statistical parameters) can be used by different machines of both datasets, due to high correlation. On the other hand, it is possible to note that Database 2 has more complexity than Database 1, due to low correlation among features (near 0). Also, Figure 9c shows the correlation for both datasets together, and, as expected, behavior characteristics related to correlation from both are presented in this one. In other words, it inherits characteristics from datasets 1 and 2, including low and high correlation complexity.

### 5.2. Machine Learning Classifiers

After creating the statistical dataset, we applied it to the machine learning framework. This is necessary to find the best classification technique, that is, that with the highest score. Also, in order to increase the classification score, we use the techniques of Feature Selection [36] and Model Tuning [37], in which the framework is able to select the best features (statistical parameters) according to the classifier and to select the best configuration from a set previously defined of that classifier.

**Figure 10:**
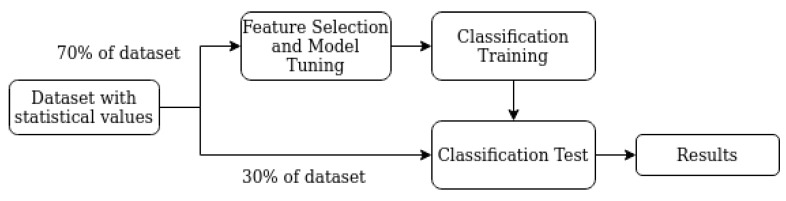
Classification Approach 1.

**Figure 11:**
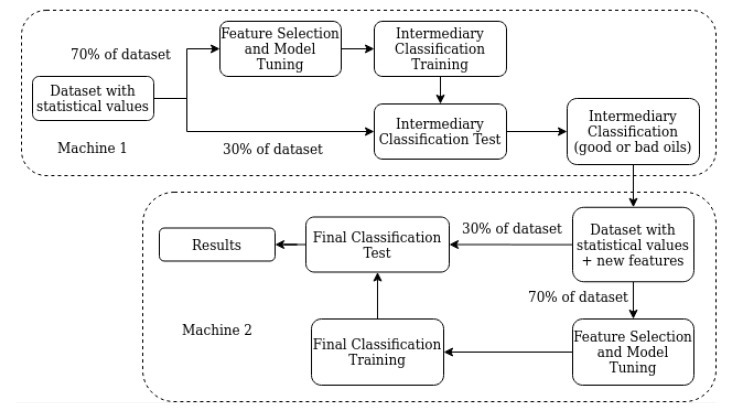
Classification Approach 2.

In addition, we developed two classification approaches according to the types of oils we had. For example, we have four types of oils—new, processed, contaminated and out of service. The first two are good oils, while the last two are not. Our first approach is to classify the statistical values from one SWEEP signal into one of the four types. While the second uses machine committee by breaking the classification in two steps. In this approach, the first machine is responsible for classifying the signal into good and bad oils and then, the second machine does the final classification. In this case, feature selection and model tuning are performed again. The output of the first machine is then utilized as a new feature by the second machine. Figure 10 and Figure 11 show, in the form of a block diagram, how the proposed framework works.

Each block of figures is detailed as follows:

- **Classification Training:** step where the chosen classifier is trained by feeding with 70% of the values from the statistical dataset. Final and Intermediary Classification Training refers to machine committee breaking classification;
- **Classification Test:** step where we feed the classifier with values that were not used in the training set, in order to test its performance;
- **Dataset with Statistical Values:** dataset with statistical values from the SWEEP signals;
- **Dataset with Statistical Values plus new features:** dataset with statistical values from the SWEEP signals and new features generated from the first classification machine (machine committee);
- **Feature Selection and Model Tuning:** step in which it is evaluated the classifiers used in this work. The techniques Grid Search Hyperparameters [37] and Cross-Validation [38] are used in conjunction to find the best classifier (with the highest score). Thus, this step helps to increase the overall system score and reduce the number of features;
- **Intermediary Classification:** step where the intermediary machine classifies the signals in good or bad oils;
- **Final Classification Training:** step where the final machine classifies the signals among one of the four types of oils (New, Processed, Contaminated, and Out of service);
- **Results:** main results are gathered in this final step.

There are a set of machine learning classifiers; however the ones employed in this work for simplicity and whose behavior are well-known among the research community include Random Forest, ExtraTree Classifier, Logistic Regression, Support Vector Machines, k-Nearest Neighbors and Stochastic Gradient Descent. In addition, we use 70% and 30% of the original dataset as the training and test set, respectively. A brief description of each classifier is presented as follows:

- **Random Forest:** it is a estimator that fits a number of Decision Tree classifiers of several sub-samples of the dataset and uses the average to improve the predictive accuracy and to control the over-fitting [39].
- **ExtraTree Classifier:** it is a learning technique very similar to Random Forest, in which it aggregates several decisions trees. However, it differentiates by using multiple de-correlated decision trees collected in a “forest” to output its classification result [40];
- **Logistic Regression:** it is a statistical model that uses a logistic function to model a binary variable. In other words, a binary logistic function or variable has only two possible values, such as yes/no. It has low implementation complexity, is suitable for linearly separable data, and is less prone to over-fitting [41];
- **Support Vector Machines (SVM)**: it is a discriminative classifier formally used to separate data between hyperplanes [42].
- **k-Nearest Neighbors (KNN):** it is a type of supervised learning technique, where the data is classified to the class most common among its *k* nearest neighbors (where *k* is a positive small integer and the neighbors are other data belonged to the same dataset) [43];
- **Stochastic Gradient Descent (SGD):** it is a very efficient approach used to find the values of coefficients of a function that minimizes a cost function, such as convex functions used in linear Support Vector Machines (SVM) and Logistic Regression [44].

## 6. Results and Analysis

This section presents and discusses our results regarding the classification of the oils, the results of Feature Selection and Model Tuning. As already presented in Figure 10 and Figure 11, we developed two classification approaches, and three datasets (also presented in Section 5.1) were applied in each of these approaches. Those results are discussed separately in Section 6.2 and Section 6.3, respectively. Firstly, the analyses of feature selection and model tuning are presented in Section 6.1.

### 6.1. Feature Selection and Model Tuning

Even though feature selection and model tuning are part of a pre-processing phase, it is also very important for the classification phase. Firstly, we perform feature selection to reduce the number of features, according to the chosen classifier. In other words, we have six different outputs of feature selection, which are applied for each classifier. In total, to decide which of the estimators have the best score to our problem, we need to perform 36 and 72 classifications in the pre-processing step, considering the classification approaches 1 and 2, respectively. Table 5 shows the average score achieved in the step of Feature Selection and Model Tuning. Note that KNN, Random Forest, and Extra Tree Classifiers have the best scores. Therefore, we have decided to use only KNN, Random Forest, and Extra Tree Classifiers as the machines at the final classification.

### 6.2. Classification Approach 1

This section presents and discusses the results regarding the first classification approach. Table 6 shows the total number of machines/classifiers with scores (ratio between correct predicted values and true labels) greater or equal to 0.98 or 0.99 or 1. Note that by using this approach, a total of 7 classifiers with a score greater or equal to 0.98 is achieved. Also, only the Datasets 1 and 3 have classifiers with 100% of the score, which means those results can achieve a perfect classification.

**Figure 12:**
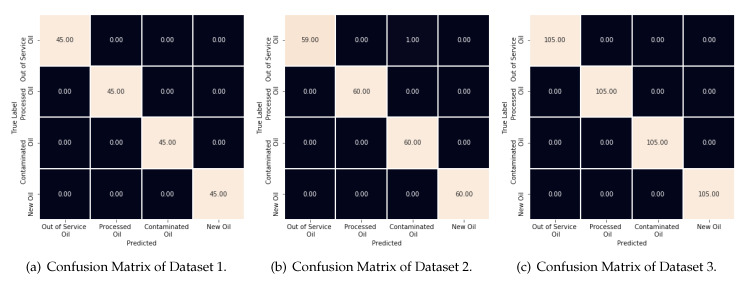
Confusion Matrice of Classification Approach 1: (**a**) Dataset 1; (**b**) Dataset 2; and (**c**) Dataset 3.

Figure 12 illustrates this behavior by showing the confusion matrices for each dataset. As one can note, the machines used in Datasets 1 and 3 were able to classify all types of oils correctly. However, the machine used in Dataset 2 has only one error (an Out of service class is selected as a Contaminated one). This is not a serious error regarding the original problem, once the two entries are the types of bad oils.

**Figure 13:**
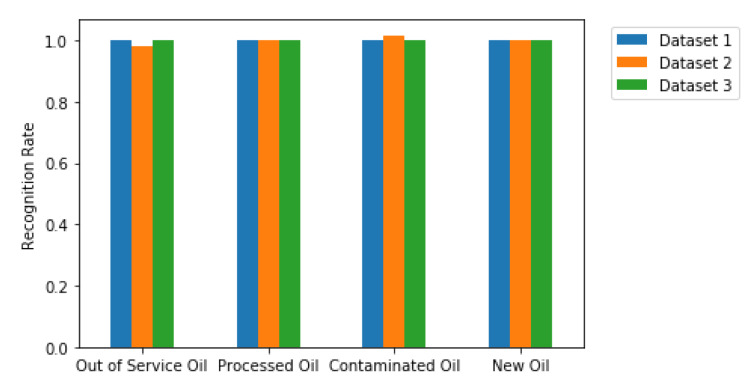
Recognition Rate per Oil and Dataset.

In addition, Figure 13 shows the score for each type of oils. Note that with the exception of 1 misprediction of contaminated and out of service oils from database 2, the rest are able to be recognized by the machines, and classified correctly.

### 6.3. Classification Approach 2

**Figure 14:**
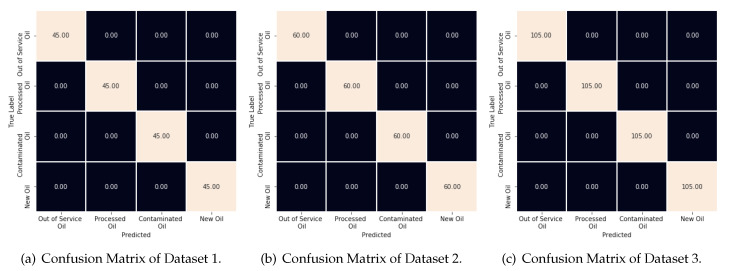
Confusion Matrices of Classification Approach 2: (**a**) Dataset 1; (**b**) Dataset 2; and (**c**) Dataset 3.

**Figure 15:**
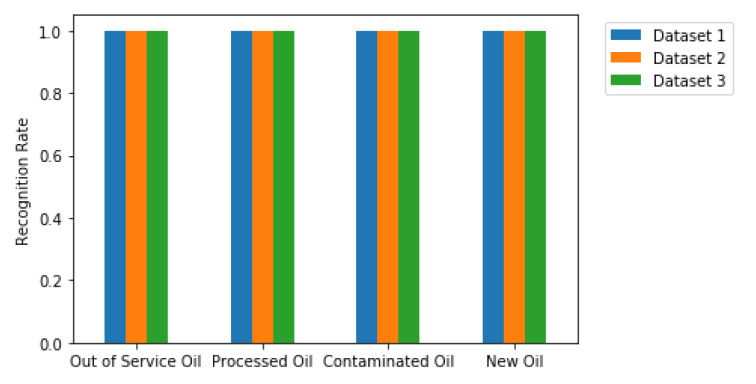
Recognition Rate per Oil and Dataset.

This section presents and discusses the results achieved by using the second classification approach. Similarly to Table 6 and Table 7 shows the total number of machines/classifiers with scores greater or equal to 0.98, 0.99 and 1. It is easy to notice an increase in the number of machines with very high scores by only using the machine committee. Thus, by selecting only one machine committee with 100% accuracy for each dataset, we generate the results shown in Figure 14 and Figure 15. The former shows the confusion matrices for all datasets analyzed, while the latter shows the recognition rate (score) per oil and dataset. Both figures show the behavior expected for these results, where it is achieved a perfect classification with no errors.

Therefore, it is important to point out that a series of steps and decisions were taken in the pre-processing phase to avoid low scores and overfitting as feature selection, model tuning with cross-validation, as already mentioned. Table 8 shows the main classification parameters for one possible configuration of machines for perfect classification, considering the approaches and the datasets involved. Note that the ExtraTree and Random Forest classifiers are the most common techniques for this problem. Moreover, considering that we were able to classify all oils available correctly, thus it is possible to integrate both classification approaches with the acoustic signal analysis from our in-home built hydrophone to create a new predictive solution for monitoring transformers.

## 7. Conclusions

In the present study, a new acoustic technique applied to the diagnosis of mineral oil used in transformers was presented. The technique integrates an in-home built thermoformed ferroelectret as an acoustic transducer and a machine learning method to support variations in the oil classification. First, we demonstrated how the transducer was built followed by the ferroelectret sensor, further some experimental results were showed and finally we integrated the collect signals into machine learning classification frameworks. Two approaches of classification were developed; in both, we were able to classify all oils available correctly. From the employed methods, we were able to obtain scores of 100% and 99% using the Extra Tree classifier from classification approach 1, and 100% with Extra Tree, the Random Forest and the k-Nearest Neighbors classifiers by using classification approach 2. The proposed method is fast compared to the physical-chemical analysis that needs measurements at energy substation followed by laboratory evaluation often located kilometers away. Our method is based on the acquisition of the signal and the classification of the oil can be made on-site in a few seconds. As future studies, we plan to feed our framework with more experimental results from oils, water with different concentrations of pollutants as well as seawater aiming to build a fast and reliable system to detect oil leakages.

## Figures and Tables

**Table 1:** Test limits for new insulating mineral oil (IMO received in new equipment or after filling, prior to energization [22].

Test	Method	Value for Voltage Class		
		≤ 69 kV	>69 kV-<230 kV	≥ 230 kV
Color ASTM units, maximum	ASTM D1500	1.0	1.0	0.5
Neutralization Number (acidity) mg KOH/g, maximum	ASTM D974	0.03	0.03	0.03
Interfacial Tension mN/m, minimum	ASTM D971	38	38	38
Dissipation Factor (Power Factor) 25 °C, % maximum	ASTM D924	0.05	0.05	0.05
Water Content mg/kg, maximum	ASTM D1533	20	10	10
Dielectric breakdown voltage kV, minimum, 1mm gap	ASTM D1816	25	30	35

**Table 2:** Physical-chemical analysis of Database 1.

Test	New_1	Processed_1	Contaminated_1	Out of service_1
Color	<0.5	0.5	1.0	3.0
Density-20/4 °C	0.878	0.880	0.872	0.866
Tension Interfacial [nN/m]	44.8	41.0	37.0	20.0
Water Content [ppm]	11.0	14.6	38.1	40.4
Neutral Index [mgKOH/g]	0.013	0.028	0.042	0.406
Electrical Breakdown Strength [kV]	63.1	60.5	16.7	17.3
Power Factor [%]	0.02	0.02	0.02	0.02

**Table 3:** Physical-chemical analysis of Database 2.

Test	New_2	Processed_2	Contaminated_2	Out of service_2
Color	<0.5	1.5	1.5	5.0
Density-20/4 °C	0.876	0.878	0.878	0.870
Tension Interfacial [nN/m]	40.3	40.0	25.4	26.0
Water Content [ppm]	8.2	11.0	46.9	31.0
Neutral Index [mgKOH/g]	0.002	0.021	0.025	0.45
Electrical Breakdown Strength [kV]	70.2	63.2	16.9	28.0
Power Factor [%]	0.02	0.02	0.02	0.03

**Table 4:** Some important characteristics of the statistical dataset.

Parameters	Value
Datasets	3
Year	2016 and 2018
Entries	180, 240 and 420
Features	84
Window	5.000 samples
SWEEP signal	100.000 samples
Statistics	Mean, Moving Mean, Variance, Moving Variance, Vpb, Moving Vpb, Correlation and Moving Correlation

**Table 5:** Average scores of studied Classifiers.

Classifiers	Mean Scores
Random Forest	0.98412698
Extra Tree	0.9984127
Logistic Regression	0.94761905
SVM	0.8
k-Nearest Neighbors	0.97619048
SGD	0.71904762

**Table 6:** Total Number of Machines According to the Score of Approach 1.

Classification Approach: #	Classifiers with Score >0.98	Classifiers with Score >0.99	Classifiers with Score = 1
1: Dataset 1	2	1	1
1: Dataset 2	1	0	0
1: Dataset 3	4	3	1
Total	7	4	2

**Table 7:** Total Number of Machines According to the Score of Approach 2.

Classification Approach: #	Comitte with Score >0.98	Comitte with Score >0.99	Comitte with Score = 1
2: Dataset 1	45	35	35
2: Dataset 2	23	9	9
2: Dataset 3	26	15	8
Total	94	59	52

**Table 8:** Classification Parameters.

Classification Approach: #	Dataset 1	Dataset 2	Dataset 3
1: Classifier 1	ExtraTreesClassifier - # of features: 38 - criterion: gini	ExtraTreesClassifier # of features: 53 - criterion: gini	ExtraTreesClassifier # of features: 47 - criterion: gini
2: Classifier 1	k-Nearest Neighbors - k neighbors: 5 - # of features: 7	ExtraTreesClassifier - # of features: 20 - criterion: gini	RandomForestClassifier - # of features: 68 - criterion: gini
2: Classifier 2	RandomForestClassifier - # of features: 33 - criterion: gini	RandomForestClassifier - # of features: 33 - criterion: gini	ExtraTreesClassifier - # of features: 63 - criterion: gini
